# Histopathologic Features of Lymphedema: A Molecular Review

**DOI:** 10.3390/ijms21072546

**Published:** 2020-04-06

**Authors:** Claire Y. Li, Raghu P. Kataru, Babak J. Mehrara

**Affiliations:** The Department of Surgery, Division of Plastic and Reconstructive Surgery, Memorial Sloan Kettering Cancer Center, New York, NY 10065, USA; lic2@mskcc.org (C.Y.L.); katarur@mskcc.org (R.P.K.)

**Keywords:** lymphedema, inflammatory skin conditions, inflammation, immunity, fibrosis, adipogenesis, recurrent infections

## Abstract

An estimated 5 million people in the United States are affected by secondary lymphedema, with most cases attributed to malignancies or malignancy-related treatments. The pathogenesis of secondary lymphedema has historically been attributed to lymphatic injury or dysfunction; however, recent studies illustrate the complexity of lymphedema as a disease process in which many of its clinical features such as inflammation, fibrosis, adipogenesis, and recurrent infections contribute to on-going lymphatic dysfunction in a vicious cycle. Investigations into the molecular underpinning of these features further our understanding of the pathophysiology of this disease and suggests new therapeutics.

## 1. Introduction

Lymphedema is a progressive and morbid disease characterized by swelling and fibrosis of the affected region resulting in functional problems, decreased quality of life, and recurrent infections. The underlying pathogenesis of lymphedema involves dysfunction in lymphatic transport, which under normal conditions is responsible for transporting residual interstitial fluid, immune cells, and lipids. Lymphedema can be further classified into primary and secondary lymphedema. Primary lymphedema is caused by a congenital dysfunction of the lymphatic system; in contrast, secondary lymphedema results from disease or iatrogenic processes. Secondary lymphedema is significantly more common and usually related to cancer treatments. Breast cancer-related lymphedema accounts for most cases of secondary lymphedema in the United States due to the high incidence of breast cancer [1]. Estimates of the risk of lymphedema following lymph node dissection for breast cancer vary and depend on the length of follow-up ranging between 16% and 60% [2,3]. The advent of sentinel lymph node sampling for breast cancer and other malignancies has drastically reduced the need for lymph node dissection; however, even sentinel lymph node biopsies involve a clinically relevant risk of lymphedema [2,4,5,6,7]. In addition to lymph node dissection, extensive skin resections, radiation therapy, obesity, and infections have also been identified as significant risk factors for disease development [4,8,9]. Lymphedema is also commonly seen in patients treated for other solid tumors, with reported incidence in melanoma of 16%, sarcoma 30%, gynecologic tumors 20%, genitourinary tumors 10%, and head and neck cancers 4% [10]. Given that the incidence of lymphedema is directly correlated with survival time after cancer therapy, the prevalence of this disease will increase as life expectancy in cancer survivors is improved with better treatments [11]. With 5 million Americans currently estimated to be suffering from this condition, lymphedema represents a great biomedical burden [11].

Currently, there is no cure or effective treatment for the management of lymphedema; the mainstay of therapy is palliative, aiming to prevent disease progression and to provide symptomatic improvements. These therapies consist of a multimodal approach known as complete decongestive therapy (CDT) and involves four components: compression therapy, manual lymphatic drainage, exercise, and skin care [12,13,14,15,16]. These palliative therapies are helpful in most patients [12,17]. However, compression therapy is usually required continuously and is a significant source of dissatisfaction among patients. Manual lymphatic drainage and physical therapy is also time consuming and expensive. This is particularly problematic since these costs are often not covered by medical insurance. Thus, developing novel therapies that resolve the underlying problem rather than simply provide symptomatic relief are an important unmet clinical need. 

More recently, surgical treatments for lymphedema such as lymphovenous bypass, vascularized lymph node transplant, and reductive techniques, including liposuction and direct excision, have been developed with varying degrees of success [18,19]. Although lymphatic surgery has proven to be helpful in some cases, these procedures inexplicably fail to improve outcomes in others, likely reflecting the complex pathophysiology of the disease [19,20]. Therefore, a greater understanding of the pathophysiology of this disease is needed to develop effective therapies. This review aims to summarize the clinical and pathologic features of secondary lymphedema and to review our present understanding regarding the molecular basis of these findings.

## 2. Clinical Presentation and Staging

The natural history of secondary lymphedema is highly variable with disease progression ranging from latent in some patients to rapidly progressive in others. Disease onset is usually insidious and delayed. Breast cancer-related lymphedema develops, on average, eight months following surgery, with 80% of patients diagnosed in the first three years after surgery [4]. The development of lower-extremity lymphedema following pelvic or inguinal lymph node dissection is more rapid, with most cases occurring 3–6 months after surgery [21,22]. Initially, affected patients may report the sensation of swelling or heaviness of the affected limb. These symptoms may lead to the development of pitting edema manifesting as an indentation in the skin after firm pressure is applied for several seconds and released. With disease progression, the skin becomes dry and firm with decreasing pitting secondary to cutaneous fibrosis and adipose deposition. The Kaposi–Stemmer sign, in which the examiner is unable to pinch the skin fold at the base of the second toe on the dorsal aspect of the foot or the webspace of the hand, is considered to be indicative of lymphedema. However, the absence of this sign is not diagnostic since in some patients with extremity lymphedema, the hands or feet are spared from swelling. Dermal thickening becomes clinically apparent at later stages of the disease and progresses to hyperkeratosis, acanthosis, lichenification, and verrucae [23]. The overlying skin may appear “cobblestoned” in appearance and is prone to fissures and recurrent infections, a hallmark of lymphedema [23]. Cellulitis, erysipelas, tinea pedis, and lymphangitis have all been associated with chronic lymphedema [23]. In very rare instances, patients with long-standing lymphedema (≥10 years) are at risk of developing an aggressive form of cutaneous angiosarcoma known as Stewart–Treves syndrome [24]. There are various classification systems used to describe disease severity, although the International Society of Lymphology (ISL) stage is the most widely used. ISL staging combines “softness” and effect of limb volume after elevation into stages 0–III. Stage 0 describes subclinical lymphedema where swelling is not evident despite impaired lymphatic transport, although patients may report a feeling of heaviness in the limb. Stage I lymphedema corresponds to limb swelling that resides with elevation or compression, usually within 24 hours. Pitting may be evident at this stage, although there is a lack of dermal fibrosis. Stage II lymphedema does not subside with elevation or compression, reflecting the consequences of dermal fibrosis. Stage III lymphedema, the most severe stage, is characterized by severe and permanent limb swelling as well as trophic skin changes such as fat deposits, acanthosis, and verrucae [23].

## 3. Role of Inflammation in Development of Lymphedema

**Figure 1:**
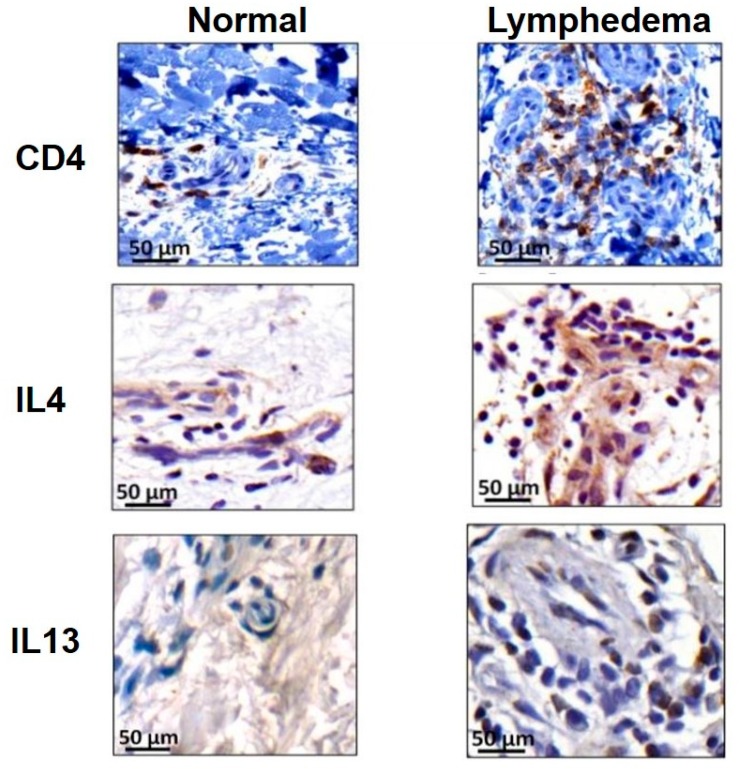
Representative immunohistochemistry images demonstrating CD4+ (upper), IL-4+ (middle), and IL-13+ (lower) cells in matched human biopsy specimens comparing lymphedematous and contralateral normal upper extremities. Reprinted with permission from supplemental figure 1 [26].

Initial lymphatic insult results in the chronic accumulation of protein-rich interstitial fluid that promotes inflammation and fibroadipose deposition. If collateral lymphatics are not able to compensate for the initial injury, these pathologic changes exacerbate persistent fluid accumulation, which further impair lymphatic dysfunction in a positive feed-back loop. Attempts to characterize the hallmark inflammatory nature of lymphedema have implicated CD4+ T cells in the inflammatory response leading to lymphedema. Avraham et al. and Zampell et al. showed that in the mouse tail surgery and popliteal lymph node dissection (PLND) models of lymphedema, greater than 70% of the inflammatory response consisted of CD4+ T cells [25,26]. Similar changes were seen in human specimens collected from patients with unilateral upper extremity breast cancer-related lymphedema, where the number of tissue-infiltrating CD4+ T cells was positively correlated with severity of disease (Figure 1) [26]. CD4-deficient mice were protected from developing lymphedema following tail lymphatic ablation; similar findings were noted when CD4+ T cells were depleted using neutralizing antibodies in wild-type (WT) mice [26]. This effect was isolated to CD4+ T cells as depletion of macrophages or CD8+ T cells did not prevent development of lymphedema [26]. Another group reported similar findings in which lymphatic vessel remodeling and collecting vessel impairment was associated with increased number of CD4+ T cells [27]. Ogata et al. found that the interaction between CD4+ T cells, specifically T helper type 1 and T helper type 17 cells, and macrophages promotes vascular endothelial growth factor C (VEGF-C) expression, which in turn led to the generation of immature and leaky lymphatic vessels that are essential for the development of initial edema [28].

Indeed, recent work from our own laboratory indicates CD4+ T cell activation is necessary and sufficient for the development of lymphedema [29,30]. Using the tail surgery and PLND models of lymphedema, Garcia Nores et al. found that adoptive transfer of CD4+ T cells to CD4-deficient mice led to the development of lymphedema similar to that seen in WT mice; in contrast, no swelling was seen in CD4-deficient mice that did not undergo adoptive transfer [29]. These authors showed that following tail surgery or PLND, CD4+ T cells are released from draining lymph nodes and home to lymphedematous skin, a process dependent on dendritic cell (DC) activation [29]. Once in the skin, CD4+ T cells promote changes such as impaired lymphangiogenesis, fibrosis, and increased inducible nitric oxide synthase (iNOS) expression leading to lymphedema [29]. Increased expression of iNOS results in disruption of the endogenous nitric oxide gradient that regulates intrinsic lymphatic pumping [31,32]. When CD4+ T cells were sequestered in the lymph node using a sphingosine-1-phosphate receptor modulator, animals did not develop lymphedema [29]. Moreover, using a mouse model of relative CD4+ T-cell deficiency created by reconstituting irradiated WT mice with CD4 knockout mouse-derived bone marrow progenitors, Ly et al. showed that even small numbers of CD4+ T cells are sufficient for the development of lymphedema and its associated histopathologic features [30].

## 4. Lymphedema as Fibrotic End-Organ Failure of Lymphatic System

Fibrosis is a common mode of end-organ failure in a variety of organ systems including the liver, lung, heart, pancreas, and kidney. Similarly, in chronic lymphedema, the collecting vessels become progressively fibrosed and are eventually replaced by scar tissue that obliterates the luminal area of the vessel [33]. In a mouse tail model of lymphedema, treatment with type I collagen gel resulted in downregulation of TGFβ-1 expression and scar formation which in turn accelerated lymphatic regeneration [34]. Collagen gel treated animals were also found to have both decreased soft tissue fibrosis and expression of pro-fibrotic genes by lymphatic endothelial cells (LECs) with concomittant improvements in lymphatic function in the form of increased number of lymphatic vessels, improved lymphatic architecture, and increased proliferation of LECs [35]. These findings suggest lymphedema may represent fibrotic organ failure of the lymphatic system.

**Figure 2:**
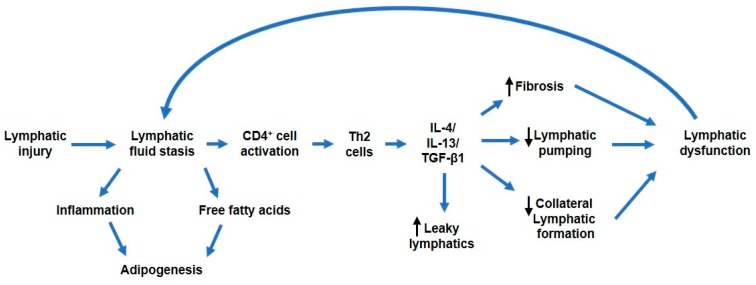
Schematic showing proposed pathophysiologic consequences following initial lymphatic injury. The accumulation of lymphatic fluid incites adipogenesis and an inflammatory response that sets off a cascade of molecular events leading to lymphatic impairment. Notably, of the inflammatory response, CD4+ T cell activation has been shown to be both necessary and sufifficent for the development of lymphedema. CD4+ T cell activation induces elaboration of T helper 2 (Th2) cytokines IL-4 and IL-13, along with transforming growth factor-β1 (TGF-β1), that contribute to lymphatic dysfunction either directly or indirectly by promoting tissue fibrosis, decreasing lymphatic pumping, impairing collateral lymphatic formation, and increasing lymphatic leakiness. The culminative effect is worsening of lymphatic fluid stasis.

Evidence from several studies highlights the importance of T helper cells in the pathogenesis of fibrosis. Notably, a T helper 1 (Th1) and T helper 2 (Th2) paradigm describes profibrotic cytokines and growth factors elaborated by Th2 cells, including IL-4, IL-13, and transforming growth factor-β1 (TGF-β1), that regulate collagen deposition and fibrosis in various organ systems [36]. For example, adoptively transferred naïve CD4+ T cells differentiated into Th1 and Th2 cells in mice that had undergone PLND [29]. These cells migrated to and proliferated in the hindlimb distal to the zone of lymphatic injury [29]. Furthermore, emerging evidence suggests a crucial role for specifically Th2 cells in the regulation of fibrosis that leads to lymphatic dysfunction [26]. Th2-deficient transgenic mice are protected from developing lymphedema and fibrosis as compared to animals with defective Th1 cell generation [37]. This finding is supported in mouse lymphedema models and human samples from breast cancer-related lymphedema, in which lymphatic injury results in a Th2-biased response with the infiltration of large numbers of CD4+ IL-4+ IL-13+ Th2 cells (Figure 1) [26,38]. Consequently, when these same animals were treated with IL-4 or IL-13 neutralizing antibodies, lymphedema was prevented [26]. The same findings were seen in a mouse asthma model in which blockade of IL-4 and/or IL-13 increased lung lymphatic vessel density and function [39]. Mechanistically, IL-4 and IL-13 downregulate LEC-specific transcription factor Prox-1 and LEC marker LYVE-1 and has been shown to impair LEC survival, proliferation, and tubule migration in vitro and in vivo (Figure 2) [40].

A large body of literature has implicated the TGF-β1 pathway as an important mechanistic player in regulating fibrosis in a number of organ systems [36,41]. Indeed, increased levels of TGF-β1 were found in lymphedematous tissue of both mice and patients, and TGF-β1 blocking antibodies resulted in both decreased fibrosis and improved lymphatic function in animal models [42,43]. The same phenomenon was observed using an animal model with dominant negative TGF-β1 receptors [42]. Interestingly, TGF-β1 blockade was also associated with decreased Th2 cell migration and expression of profibrotic Th2 cytokines (Figure 2) [42]. This reciprocal interaction between TGF-β1 signaling and Th2 cell response is also consistent with the development of fibrosis in other organ systems [44]. Although the exact mechanism by which fibrosis induces lymphatic dysfunction requires additional clarification, it is likely the result of progressive obliteration of the initial and collecting lymphatic vessels and impaired collateral regeneration [45].

## 5. Lymphedema Results in Adipose Deposition

Adipose deposition is a late stage pathologic feature of lymphedema. Several studies provide evidence that lymphatic fluid stasis drives adipose differentiation. Prox-1 is a master regulator of lymphatic development, and Prox-1 knockout mice develop chylothorax and die in utero or shortly after birth [46]. Mice with heterozygous inactivation of Prox-1 survive in some genetic backgrounds and become obese as adults even when fed a normal chow diet [47]. In these animals, fat deposition occurs most abundantly around the abnormal mesenteric lymphatics [48]. More recent studies have shown that fatty acids in lymphatic fluid promote adipocyte proliferation and differentiation, suggesting that lymphatic stasis can directly increase adipose deposition in affected tissues [49]. Consistent with these findings, Zampbell et al. and Aschen et al. found that adipose differentiation markers, including adiponectin and CCAAT/enhancer-binding protein-alpha, are increased following lymphatic injury in a mouse model of lymphedema [50,51]. IL-6, a known regulator of adipose tissue homeostasis, appears to be a negative regulator of adipose deposition in the setting of lymphedema as loss of IL-6 function resulted in increased adipose deposition in the tail surgery model of lymphedema in one study [52]. Interestingly, in the same study, it was shown that IL-6 is increased locally and systemically in patients with lymphedema, which was corroborated in animal models [52]. These data suggest IL-6 plays an important homeostatic role to limit adipose deposition in response to lymphatic injury and inflammation [52].

Given that obesity is a well-recognized risk factor for the development of secondary lymphedema, it is not suprising that on a molecular level, the relationship between lymphatic dysfunction and adipogenesis appears bidirectional [2,53]. In vitro, LECs exposed to long-chain free fatty acids have increased markers of cell death and decreased lymphatic-specific genes [49]. Similarly, LECs from obesity-prone mice showed decreased lymphatic markers compared to obesity-resistant animals [49]. Furthermore, not only did obese mice have impaired baseline lymphatic dysfunction, but they also exhibited heightened inflammatory responses, increased adipose deposition, and increased fibrosis in the setting of lymphatic injury compared to their lean counterparts [38]. Detmar and colleagues reported similar associations between obesity and lymphedema in mice with high-fat diet-induced obesity in which adiposity was associated with impaired collecting lymphatics [54]. This effect on lymphatic function was independent of lipid exposure as high-fat diet in the absence of obesity did not worsen lymphedema [55].

The relationship between obesity and lymphatic dysfunction have been demonstrated in several human studies as well. Arngrim et al. found that obese individuals have decreased adipose tissue lymphatic clearance of macromolecules when compared with lean subjects [56]. In fact, several studies have shown that some very obese patients spontaneously develop lower extremity lymphedema even without antecedent injury [57]. In a retrospective study of 51 patients with lower extremity swelling, patients with a body mass index (BMI) ≥ 30kg/m^2^ were more likely to have abnormal lymphoscintigrams findings as compared with patients with a BMI <30kg/m^2^ [58]. In this study, a threshold BMI of 50kg/m^2^ was identified as predictive of an abnormal lymphoscintigram result [58]. The mechanism of obesity-induced lymphatic dysfunction is not well understood; one possibility is that increased lymphatic fluid in an enlarging limb overwhelms the draininage capacity of the existing lymphatic system. However, evidence from animal studies supports the hypothesis that obesity-induced lymphatic injury is more than just overflooding of the lymphatic system from increased lymph. Additional cellular mechanism likely play a role. Indeed, obesity in mouse models results in a perilymphatic accumulation of inflammatory cells. These inflammatory cells likely contribute to the pathology of obesity-induced lymphatic dysfunction since inhibition of inflammatory reactions with topical IL-2 inhibitors or genetic deficiency of CD4+ T cells decreased the severity of lymphatic injury in obese animals [59]. Given the link between obesity and lymphedema, several clinical trials have investigated the potential therapeutic benefit of weight loss on breast cancer-related lymphedema and have reported mixed results. While a pilot clinical trial of 21 patients showed significant differences in limb volume with weight loss, larger trials, including the PAL and WISER trials, failed to replicate these findings [60,61,62]. However, both the PAL and WISER trials had a follow-up time of 1 year, which may not be sufficient time to observe the true effects of weight loss given the chronic course of lymphedema. Differences in follow-up time, method of limb volume measurements, and sample size likely contributed to differences in these studies, and additional investigations into the long-term effects of weight loss on lymphedema is warranted. 

## 6. Lymphedema Is Associated with Recurrent Infections

About a third of patients with lymphedema develop recurrent soft tissue infections [63,64]. These infections often require hospitalization for intravenous antibiotics and can be severe in some cases [63,65]. Indeed, some patients require lifelong suppressive antibiotic therapy and develop infections despite these treatments. Often, infections lead to increasing severity of lymphedema, suggesting that infections can injure the lymphatic system [66]. In some patients, infections are cited as a cause of secondary lymphedema, suggesting a direct role of bacteria in generating lymphatic dysfunction [67,68]. This concept is supported by a recent study demonstrating the interplay between bacteria and the lymphatic system on a molecular level. Jones et al. showed that when mice are locally inoculated with Methicillin-resistant *Staphylococcus aureus* (MRSA) in the hindlimb, they developed lymphatic dysfunction that was sustained even after the infection and inflammation had resolved [69]. Lymphatic dysfunction in these animals was associated with the loss of lymphatic muscle cells (LMCs), a critical component for lymphatic contraction [69]. In vitro experiments using MRSA-conditioned media showed that bacterial products can cause LMC death [69]. The causative agents for this injury were noted to be exotoxins controlled by gene regulator *agr*; notably, infection with *agr* mutant MRSA did not reduce lymphatic function or cause LMC injury [69]. 

Interestingly, several studies also demonstrated the detrimental effects of lymphatic dysfunction on host humoral and innate immunity as well as peripheral tolerance. The accumulation of T-regulatory cells (Tregs) at sites distal to lymphatic injury impairs bacterial phagocytosis, DC activation, antibody production, and T-cell mediated inflammation in response to lymphatic injury [70]. Depletion of Tregs restored these immunologic responses [70]. Conversely, Tregs also play a role in suppressing the hallmark inflammatory response necessary for the development of lymphedema; transgenic mice with an inducible form of Treg depletion sustained worsening edema associated with increased inflammatory infiltrate [71]. Not surprisingly, increased infiltration of Tregs has been seen in both mouse and human lymphedematous tissues [70,71]. These findings suggest dual roles of Tregs in lymphedema that involve dampening of the inflammatory response that contributes to lymphatic dysfunction as well as dampening of the host humoral and innate immunity.

Additional impairments in host immunity have been reported in both obese and transgenic mice with primary lymphatic dysfunction. In mice with obesity-induced lymphatic dysfunction, heightened dermatitis was seen in response to inflammatory skin stimuli that was reversible with treatment to promote lymphangiogenesis [38]. Similar alterations in peripheral tolerance were noted in K14-VEGFR-3-Ig mice that lack dermal lymphatics; these animals demonstrated robust contact hypersensitivity that became pathologic at 1 year when signs of autoimmunity became apparent [72]. K14-VEGFR-3-Ig mice were also found to have decreased antibody titers in response to dermal immunization, a phenomenon attributed to impaired antigen transport and DC trafficking that was seen in these animals, since B cells isolated from transgenic mice demonstrated similar functionality compared to those of WT mice [72]. Similarly, in an inducible mouse mode of local lymphatic ablation, lymph node transplant improved lymphedema as well as DC trafficking and the adaptive immune response [73]. Taken together, these results suggest an interconnected role of the lymphatics system and infection in which microorganisms contain innate mechanisms to damage host lymphatics; at the same time, lymphatic dysfunction can impair the host immune response.

## 7. Future Directions and Conclusion

Lymphedema is a chronic, debilitating, and currently incurable disease. The delayed presentation and variable natural history of lymphedema along with the observation that disease development is only seen in a subset of patients with lymphatic injury suggests additional pathologic events are necessary to tip the balance against effective lymphangiogenesis. Recent evidence suggests inflammation, fibrosis, adipose deposition, and even infectious etiologies in the setting of initial lymphatic insult is responsible for maintaining the vicious cycle of lymphatic dysfunction. Investigations into the molecular underpinnings of how lymphedema contributes to these pathologic features and vice versa suggests new strategies aimed at disease prevention and treatment. To this aim, animal studies have contributed significantly to a greater understanding of the pathogenesis of lymphedema and its pathologic findings. However, no animal model is perfect, and each has its limitations. The tail surgery and PLND mouse models of lymphedema are commonly used, however both represent acute models of lymphedema that spontaneously resolve over time with little fibrosis or adipose deposition. Our laboratory recently reported a new mouse model with an inducible mechanism of non-surgical LEC ablation, resulting in chronic and progressive edema with similar histologic and radiographic features as seen in the human disease [74]. Concurrent analysis with clinical specimens in addition to the utilization of various mouse models corroborates and strengthens the findings of preclinical studies. Indeed, several surgical and pharmacologic modalities to treat lymphedema have been tested as a result of animal studies such as lymph node transplant, stem cell therapy, and drugs to target inflammation (ketoprofen, ubenimex) and to promote lymphangiogenesis [75,76].

Alitalo and colleagues first tested adenovirally delivered VEGF-C in mouse models of lymphedema in which the axillary lymph node was surgically excised [77]. In these animals, short-term treatment with VEGF-C resulted in leaky lymphatics at 2 weeks without improvements in drainage. These leaky vessels were associated with a discontinuous pattern of adherens and tight junction proteins as well as non-functioning valves [77]. However, by 6 months, collecting lymphatic leakiness decreased with concomitant increased uniformity of endothelial junctions, functionality of vessel valves, and improved drainage [77]. Despite successful regeneration of collecting lymphatic vessels, lymph node regeneration did not occur [77]. To accomplish this, VEGF-C therapy was combined with lymph node transplantation, which resulted in increased incorporation of transplants into the existing lymphatic vasculature and improved drainage compared to control animals [77]. Of note, VEGF-C is upregulated in lymphedematous tissue, suggesting that the formation of collateral lymphatic vasculature is inhibited in this setting [78]. The exact mechanism for this phenomenon requires additional investigation but may be attributed to the persistence of fibrotic tissue at the zone of injury or lack of normal lymphatic vessel anatomy; consequently, it is possible that the combination of VEGF-C treatment with lymph node transplantation is necessary for the development of functional collateral lymphatic circulation. The therapeutic benefit of combined VEGF-C and lymph node transplant was additionally shown in a porcine mode of lymphedema [79]. These animal studies led to the patent of AdAptVEGF-C Adenoviral Vector, which is currently under active clinical testing [80].

Our understanding of the role of fibrosis, adipogenesis, and bacterial infections in the pathogenesis of lymphedema is still yet emerging and there remains a gap in clinical translation. However, discovery of the inflammatory nature of lymphedema has led to the clinical testing of various anti-inflammatory drugs, albeit with varying success. Ketoprofen, a nonsteroidal anti-inflammatory drug, showed promise in the mouse model of lymphedema in which treatment improved swelling and histopathologic changes seen in lymphedematous animals compared to controls [81]. However, in an open-label clinical trial, there was no difference between ketoprofen and placebo in regards to limb volume, although there were improvements in skin thickness and histopathology in the ketoprofen treatment arm [76]. Subsequent preclinical studies showed that the mechanism of ketoprofen’s therapeutic effect on lymphedema was due to its inhibition of the 5-lipoxygenase metabolite leukotriene B_4_ (LTB_4_) [75]. Antagonism of LTB_4_ improved lymphedema by improving lymphatic function and restoring lymphatic networks in the mouse tail model of lymphedema [75]. These findings led to the clinical testing of ubenimex, a protease inhibitor that antagonizes the biosynthesis of LTB_4_, among several other targets. Unfortunately, ubenimex also failed to demonstrate improvements in limb volume in a phase 2 clinical trial [82]. Our laboratory has found that topical tacrolimus, a T-cell immunosuppressive drug, decreases lymphedema in association with decreased T-cell infiltration and tissue fibrosis without systemic absorption in the mouse tail model of lymphedema; this drug may hold promise for future clinical trials [83]. 

The pathogenesis of secondary lymphedema has proven to be more complex than simple lymphatic injury. Common pathologic features seen in lymphedema are not mere manifestations of the disease but also contribute to its pathogenesis. These molecular interactions between lymphedema and its various pathologic findings represent potential vulnerabilities for which new pharmeutical therapies may be developed.

## Abbreviations

CDT	complete decongestive therapy
ISL	International Society of Lymphology
PLND	popliteal lymph node dissection
WT	wild-type
VEGF-C	vascular endothelial growth factor C
DC	dendritic cell
iNOS	inducible nitric oxide synthase
LEC	lymphatic endothelial cell
Th1	T helper 1
Th2	T helper 2
TGF-β1	transforming growth factor-β1
BMI	body mass index
MRSA	Methicillin-resistant *Staphylococcus aureus*
LMC	lymphatic muscle cell
Treg	T-regulatory cell
LTB_4_	leukotriene B_4_
